# Serum carboxypeptidaseA4 levels predict liver metastasis in colorectal carcinoma

**DOI:** 10.18632/oncotarget.12798

**Published:** 2016-10-21

**Authors:** Lichao Sun, Chunguang Guo, Joseph Burnett, Zhihua Yang, Yuliang Ran, Duxin Sun

**Affiliations:** ^1^ State Key Laboratory of Molecular Oncology, National Cancer Center/Cancer Hospital, Chinese Academy of Medical Sciences and Peking Union Medical College, Beijing, 100021, P.R. China; ^2^ Department of Pharmaceutical Sciences, University of Michigan, Ann Arbor, MI 48109, USA; ^3^ The Department of Abdominal Surgical Oncology, Cancer Hospital, Chinese Academy of Medical Sciences (CAMS), Beijing 100021, P.R. China

**Keywords:** CPA4, marker, liver metastasis, colorectal cancer, prognosis

## Abstract

Hepatic metastasis is the most critical prognostic factor for colorectal cancer (CRC), and early detection of CRC liver metastasis can significantly improve cancer patient outcomes. In this study, we examined the levels of CPA4 in CRC samples, and assessed the potential of serum CPA4 as a biomarker for predicting CRC liver metastasis. CPA4 positivity was observed in 68.4% (130/190) colorectal cancer tissues, and significantly correlated with Depth of invasion, Lymph node metastasis, Distant metastasis and Stage. In addition, high CPA4 expression was associated with poor overall survival, and was an independent prognostic marker in patients with CRC. In CRC serum samples, serum CPA4 concentrations in CRC-M1(S) patients (3717.89 ± 375.98 pg/mL) were significantly increased as compared to in CRC-M1(H) patients (3692.12 ± 261.51 pg/mL), CRC patients without liver metastasis (2480.47 ± 507.90 pg/mL) or healthy controls (2183.7 ± 621.7 pg/mL) (*P* < 0.05). Furthermore, high CPA4 concentration was significantly correlated with Distant metastasis, Lymph node involvement, Stage and poor overall survival of the patients with CRC. Logistic regression analysis revealed that serum CPA4 level and Lymph node metastasis were the significant parameters for predicting CRC liver metastasis. In leave-one-out-cross-validation, these two markers resulted in sensitivity (90.0%) and specificity (93.8%) for hepatic metastasis detection. Moreover, this combination could correctly classify 49 cases of the 50 CRC patients with heterochronous liver metastasis in an independent test set. Therefore, our results suggest that CPA4 is closely associated with CRC liver metastasis, and serum CPA4 concentration combined with lymph node involvement may be used as accurate predictors of liver metastasis in colorectal cancer.

## INTRODUCTION

Colorectal carcinoma (CRC) is one of the leading causes of cancer death worldwide and prone to liver metastasis [1]. It is estimated that 15–25% of the CRC patients have liver metastases at the time of diagnosis (synchronous), and another about 20% ones would develop liver metastasis after resection of the primary tumor (heterochronous) [2, 3]. Liver metastasis is the most critical prognostic factor for CRC. Thus, early detection of liver metastasis is important for improving the survival for CRC patient.

Carboxypeptidase A4 (CPA4) is a member of the metallocarboxypeptidase family, and is aberrantly overexpressed in some types of cancer tissues [4, 5]. As a secreted proteinase, CPA4 was closely associated with the establishment of cancer microenvironment to facilitate cancer progression. Previous studies in our lab indicated that CPA4 level was significantly elevated in pancreatic cancer tissues as well as serum samples, and it might be a potential diagnostic serum marker for pancreatic cancer [6]. Moreover, elevated expression of CPA4 was observed in Non-small-cell lung cancer (NSCLC) samples, and serum CPA4 level combining with CYFRA21-1 could be used to aid early detection of NSCLC [7]. CPA4 has been implicated in prostate cancer aggressiveness [8]. Until now little is known about the expression of CPA4 in colorectal carcinoma tissues and its predictive value for liver metastasis.

In this study, we examined CPA4 level in primary colorectal cancer tissue and serum samples, and analyzed the correlation between CPA4 levels and clinicopathological characteristics of CRC patients. We propose that serum CPA4 might be a candidate diagnostic biomarker for colorectal cancer liver metastasis.

## RESULTS

### CPA4 expression and its association with pathological characteristics in CRC tissues

**Figure 1:**
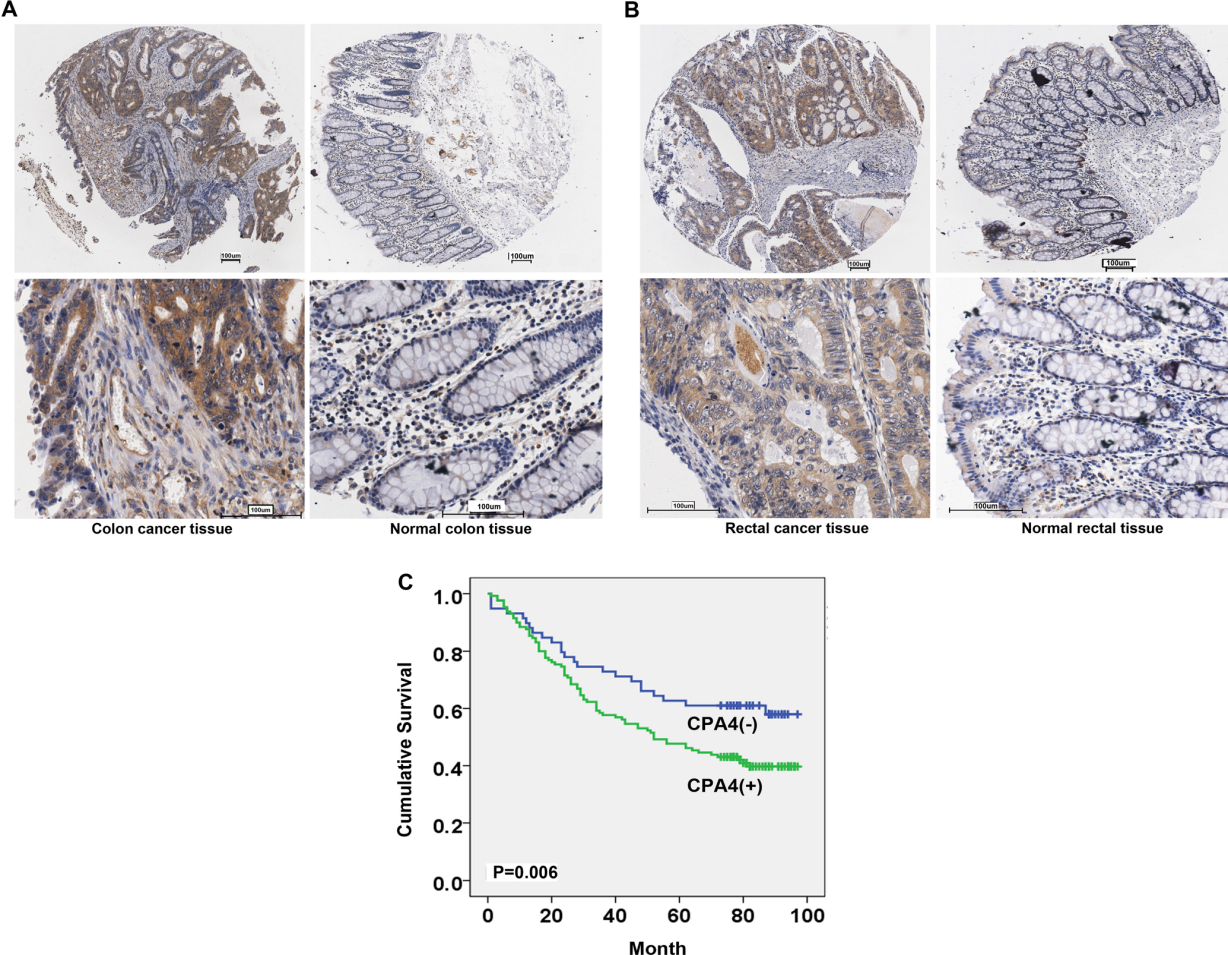
Expression of CPA4 in human primary colorectal cancer and normal tissues (**A**) Representative immunohistochemical staining of CPA4 in colon cancer samples and matched normal tissues. (**B**) Representative immunohistochemical staining of CPA4 in rectal cancer samples and matched normal tissues. (scale bar, 100 μm). (**C**) Kaplan-Meier survival curve of colorectal cancer patients with CPA4 expression in tissues. Patients with positive CPA4 expression showed significantly lower survival rates. (*P* = 0.006).

CPA4 positivity was observed in 68.4% (130/190) CRC cases. There was weak or no staining of CPA4 in the normal colorectal epithelium. Also, CPA4 positive staining was found in interstitial tissues especially around micro-vessels areas in CRC tissues (Figure 1A, 1B). Further analysis revealed that CPA4 expression was significantly associated with depth of invasion (*P* = 0.023), lymph node metastasis (*P* = 0.043), distant metastasis (*P* = 0.045) and clinical stage (*P* = 0.022) (Table 1). Importantly, the positive rate of CPA4 CRC patients with distant metastasis was 100%. While there was no significant correlation between CPA4 expression and tumor size, differentiation, age and Gender. Taken together, these observations indicated that CPA4 might play a key role in the cancer metastasis.

**Table 1:** Correlation between CPA4 level and clinicopathological characteristics in 190 colorectal cancer tissues

	CPA4 Level		*p*-value
	negative	positive	
Gender (male:female)	34:26	79:51	0.592
Age	64.17 ± 20.54	63.81 ± 15.45	0.894
Type			0.622
Colon	30	70	
Rectal	30	60	
Tumor size (cm)			0.161
> 5 cm	37	66	
≤ 5 cm	23	64	
Differentiation			0.709
Well + Moderate	49	109	
Poor	11	21	
Depth of invasion			0.023*
T1 + T2	12	11	
T3 + T4	48	119	
Lymph node metastasis			0.038*
N0	41	68	
N1 + N2	19	62	
Distant metastasis			0.045*
M0	60	122	
M1	0	8	
Grade			0.709
high + moderate	49	109	
low	11	21	
Stage			0.022*
I	9	7	
II	32	61	
III	19	54	
	0	8	
Location			0.579
Right-sided colon cancer	13	37	
Left-sided colon cancer	17	31	
Rectal cancer	30	62	

### The correlation of CPA4 expression with prognosis of colorectal cancer

The prognostic significance of CPA4 expression was determined by CPA4 staining and the corresponding clinical follow-up records. Kaplan-Meier survival analysis revealed a correlation between higher CPA4 expression levels and shorter overall survival times (*P* = 0.006) (Figure 1C). Further Cox multivariate regression model demonstrated that CPA4 level (HR = 1.780; 95% CI: 1.090–2.908; *P* = 0.021), Grade (HR = 2.267; 95% CI: 1.369–3.725; *P* = 0.001), Distant metastasis (HR = 3.207; 95% CI: 1.121–9.179; *P* = 0.030),and lymph node metastasis (HR = 3.428; 95% CI: 1.565–7.509; *P* = 0.002) were statistically independent predictive factors of poorer prognosis for CRC cancer (Table 2).

**Table 2:** Multivariate analysis of cox proportional hazards model for colorectal cancer tissue samples

Characteristics	B	SE	Wald	df	Sig.	Exp (B)	95.0% CI for Exp (B)	
							Lower	Upper
Location (right sided colon cancer vs.Left sided colon cancer+Rectal cancer)	−.241	.233	1.066	1	.302	.786	.497	1.242
CPA4	.577	.250	5.307	1	.021	1.780	1.090	2.908
Grade	.818	.257	10.127	1	.001	2.267	1.369	3.752
Depth of invasion	.586	.372	2.485	1	.115	1.797	.867	3.725
Lymph node metastasis	1.232	.400	9.482	1	.002	3.428	1.565	7.509
Distant metastasis	1.165	.537	4.719	1	.030	3.207	1.121	9.179
Stage	−.324	.330	.967	1	.325	.723	.379	1.380

### Clinical significance of serum CPA4 level in colorectal cancer

**Figure 2:**
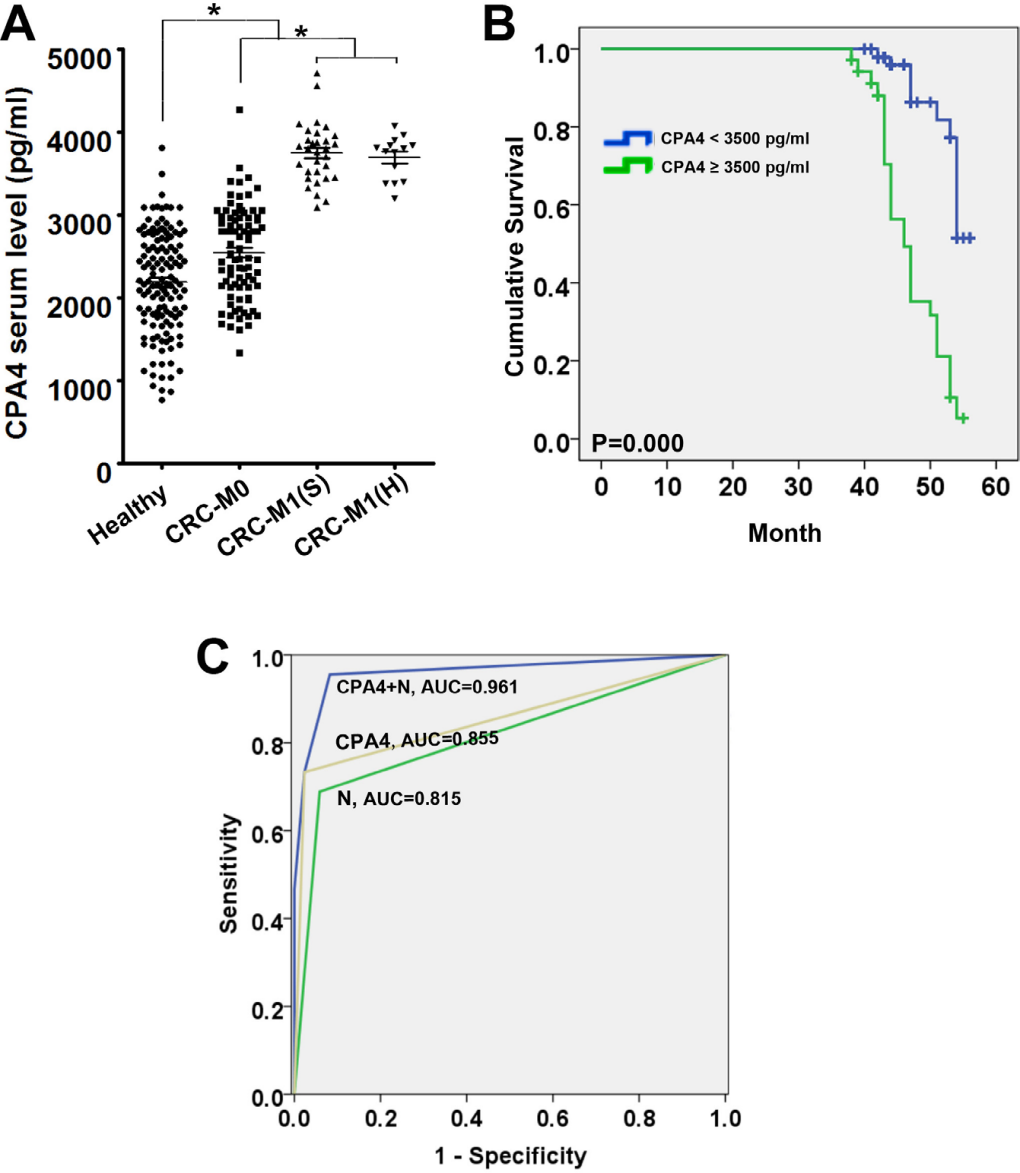
Estimated overall survival according to the expression of CPA4 in tissues and serum samples of colorectal cancer (CRC) (**A**) The serum levels of CPA4 were measured by ELISA in healthy people, colorectal cancer patients without liver metastasis, M1(S), and M1(H). (**B**)The receiver operating characteristics curves and the corresponding values of area under the curve (AUC) of CPA4, N (lymph node involvement) and in combination in the training set. (**C**) Kaplan-Meier survival curve of colorectal cancer patients with CPA4 level in serum. Patients with high level of CPA4 showed significantly lower survival rates. (*P* = 0.000).

To investigate the potential of CPA4 as a serological marker for predicting CRC liver metastasis, we firstly measured the CPA4 levels in CRC serum samples (*n* = 130) by ELISA. The clinicopathological characteristics of CRC patients was shown in Table 3. The results demonstrated that serum concentrations of CPA4 in CRC patients (2953.6 ± 751.2 pg/mL) were significantly higher than those in healthy controls (2183.7 ± 621.7 pg/mL) (*P* < 0.05). Further analysis indicated that the serum levels of CPA4 were 2480.47 ± 507.90 pg/mL in colorectal cancer without liver metastasis, 3717.89 ± 375.98 pg/mL in colorectal cancer with simultaneous liver metastasis, and 3692.12 ± 261.51 pg/mL in colorectal cancer with heterogeneous liver metastasis and the differences were significant (*p* < 0.05) (Figure 2A). The clinical profile of colorectal cancer patients with a serum CPA4 level above the cut-off level (3500 pg/mL) was shown in Table 4. High serum CPA4 levels were significantly correlated with Distant metastasis (*p* = 0.000), Lymph node involvement (*p* = 0.000), Stage (*p* = 0.000), serum CEA level (*p* = 0.015), Serum TPS level (*p* = 0.004) and Serum CA199 level (*p* = 0.001). Kaplan-Meier survival analysis showed that increased CPA4 concentrations of 3500 pg/mL or higher in colorectal cancer serum were correlated with poor overall survival (*P* = 0.000) (Figure 2B). Further Cox multivariate regression analysis revealed that elevated serum CPA4 level (HR = 2.175; 95% CI: 1.272–3.719; *P* = 0.005), Serum CA199 level (HR = 2.244; 95% CI: 1.201–4.194; *P* = 0.011), Stage (HR = 3.502; 95% CI: 1.418–8.650; *P* = 0.007) and Distant metastasis (HR=1.629; 95% CI: 1.023–2.593; *P* = 0.040) were independent risk factor for reduced survival in CRC cancer patients (Table 5).

**Table 3:** Clinicopathological characteristics of the 130 serum colorectal cancer samples

	CRC-M0	CRC-M1(S)	CRC-M1(H)	*P*
Gender (male:female)	50:35	21:10	9:5	0.666
Age	58.62 ± 12.13	59.06 ± 9.44	59.21 ± 10.17	0.972
Differentiation				0.055
Well + Moderate	78	25	10	
poor	7	6	4	
Depth of invasion				0.362
T1 + T2	31	8	3	
T3 + T4	54	23	11	
Lymph node metastasis				0.000*
N0	79	11	4	
N1 + N2	6	20	10	
Stage				0.000*
I	33	0	2	
II	48	0	2	
III	4	0	10	
IV	0	31	0	
Location				0.675
Right-sided colon cancer	16	4	2	
Left-sided colon cancer	21	10	2	
Rectal cancer	48	17	10	
Serum CPA4 level				0.000*
negative	83	8	4	
positive	2	23	10	
Serum CEA level				0.000*
negative	63	6	9	
positive	22	25	5	
Serum CA199 level				0.000*
negative	81	11	14	
positive	4	20	0	
Serum TPS level				0.000*
negative	68	7	10	
positive	17	24	4	

**Table 4:** Correlation between serum CPA4 level and clinicopathological characteristics in 130 cases

	CPA4 Serum Level		
	negative	positive	*p*-value
Gender (male:female)	57:38	23:12	0.553
Age	58.13 ± 12.06	60.6 ± 8.67	0.269
Differentiation			0.155
Well + Moderate	85	28	
Poor	10	7	
Depth of invasion			0.580
T1 + T2	32	10	
T3 + T4	63	25	
Lymph node metastasis			0.000*
N0	80	14	
N1 + N2	15	21	
Distant metastasis			0.000*
M0	83	2	
M1 (synchronous)	8	23	
M1 (heterogeneous)	4	10	
Stage			0.000*
I	33	2	
II	47	3	
III	7	7	
IV	8	23	
Location			**0.764**
Right-sided colon cancer	17	5	
Left-sided colon cancer	25	8	
Rectal cancer	53	22	
Serum CEA level			0.015*
Negative	63	15	
Positive	32	20	
Serum TPS level			0.004*
Negative	69	16	
Positive	26	19	
Serum CA199 level			0.001*
Negative	84	22	
Positive	11	13	

**Table 5:** Multivariate analysis of cox proportional hazards model for colorectal cancer serum samples

Characteristics	B	SE	Wald	df	Sig.	Exp(B)	95.0% CI for Exp (B)	
							Lower	Upper
Location (right sided colon cancer v.s. Left sided colon cancer + Rectal cancer)	−.234	.328	.508	1	.476	.792	.416	1.506
Chemothreapy(with ot without)	.419	.281	2.225	1	.136	1.520	.877	2.637
Depth of invasion	.475	.351	1.837	1	.175	1.608	.809	3.197
Lymph node metastasis	−.694	.444	2.449	1	.118	.500	.209	1.191
Stage	1.253	.461	7.379	1	**.007**	3.502	1.418	8.650
CA199	.808	.319	6.418	1	**.011**	2.244	1.201	4.194
TPS	.373	.265	1.981	1	.159	1.453	.864	2.443
CEA	−.057	.353	.026	1	.871	.944	.472	1.887
CPA4	.777	.274	8.060	1	**.005**	2.175	1.272	3.719
Grade	.201	.326	.381	1	.537	1.223	.646	2.317
Distant metastasis	.488	.237	4.229	1	**.040**	1.629	1.023	2.593

### The CPA4 levels can predict the risk of colorectal cancer liver metastasis

Logistic regression was done to calculate the respective significance of each candidate marker and clinical features for predicting liver metastasis. Both the CPA4 level and Lymph node metastasis showed predictive significance (*P* < 0.05) and increased the metastatic ratio. The odds ratios of CPA4 and Lymph node metastasis were 181.44 and 30.677, respectively (Table 6). To further evaluate the predictors that reached statistical significance in logistic regression analysis, the two variables were selected for leave-one-out-cross-validation analysis. This analysis revealed that CPA4 and Lymph node metastasis yielded the most satisfactory sensitivity (95.6%) and specificity (91.8%). We next calculated the receiver operating characteristic curve for the combination in training set. The area under the curve for the combination of serum CPA level and N (lymph node involvement) was 0.961. (Figure 2C). Meanwhile, we also got a discriminant equation for the prediction of CRC liver metastasis in an independent test set (*n* = 50). Moreover, of the 50 heterochronous metastatic cases, this combination could correctly classify 49 cases.

**Table 6:** Multiple logistic regression analysis of serum markers and clinical parameters

Variable	Regression coefficient	*P* value	Odds ratio	Confidence intervals
Serum CPA4 level	5.201	0.000	181.440	18.537–1775.913
Serum CA199 level	1.830	0.100	6.231	0.703–55.198
Serum TPS level	0.579	0.500	1.784	0.332–9.591
Serum CEA level	0.102	0.903	1.108	0.213–5.746
Lymph node metastasis	3.424	0.000	30.677	6.330–148.671

## DISCUSSION

Colorectal carcinoma (CRC) is one of the major causes of cancer death throughout the world [9]. The presence of hepatic metastases is the most critical prognostic factor for patients with CRC [2, 10]. It is estimated that about 15–25% CRC patients have synchronous liver metastases, and other 20% ones would develop heterochronous liver metastases after resection of the primary tumor. Clinically, CRC patients with liver metastases would be recommended to remove the primary and liver metastatic lesion if the patients have liver-limited metastatic foci [11]. However, CRC patients with heterochronous liver metastases should be treated with chemotherapy (FOLFOX4) or targeted therapy (Cetuximab) before and after surgery, if they are resectable [12]. Therefore, the early detection of liver metastasis could significantly contribute to treatment options and improve the survival of CRC patients.

It has been reported that a great number of serum biomarkers including CEA, CA199 and TPS could be used for early diagnosis and monitoring of CRC liver metastasis [13]. A single assay for each of them hardly achieved high sensitivity and specificity for early detection of liver metastasis. Although combination of CEA, CA199 and TPS could enhance their efficiency in diagnosis of liver metastases, the results of predictive ability were not greatly consistent [14, 15]. More importantly, high levels of CEA, CA199 and TPS were also observed in other types of cancer including gastric cancer and pancreatic cancer or inflammatory lesions [16–18]. Therefore, it is necessary to identify new biomarkers for early detection of CRC liver metastasis.

Carboxypeptidase A4 (CPA4) is a secreted, zinc-dependent metallocarboxypeptidase that removes the C-terminal amino acid residues of proteins [4]. Aberrant expression of CPA4 was found in some types of cancer tissues, and it could be measured as cancer markers for early detection. In prostate cancer, CPA4 gene was reported to be imprinted and may be closely associated with cancer aggressiveness [19]. Recent studies in our lab also indicated that CPA4 level was significantly elevated in pancreatic cancer tissues as well as serum samples, and was closely correlated with tumor progression. In NSCLC, CPA4 was highly overexpressed and was associated with an unfavorable prognosis. Moreover, serum CPA4 level combining with serum CYFRA21-1 level might be a diagnostic biomarker of NSCLC [7]. Despite the important clinical significance of CPA4 in many types of cancer, no previous studies have examined the level of CPA4 in colorectal cancer tissue and serum samples, and studied its predictive value for liver metastasis.

In this study, we firstly evaluated the expression of CPA4 in two different commercial tissue arrays containing 100 cases of colon cancer patients and 90 cases of rectal cancer patients with follow-up record. The results indicated that high CPA4 expression was observed in 68.4% (130/190) colorectal cancer samples, and significantly correlated with depth of invasion, lymph node metastasis, distant metastasis and Stage. Importantly, we found that the positivity of CPA4 in CRC primary tissues with distant metastasis was 100%. Kaplan-Meier survival analysis revealed a correlation between higher CPA4 expression levels and shorter overall survival times. Further Cox multivariate regression model demonstrated that CPA4 level was an independent predictive factors of poorer prognosis for CRC cancer. Taken together, these observations indicated that CPA4 might play a key role in the distant metastasis.

To further test the efficacy of CPA4 as a serum marker for CRC liver metastasis, we collected 180 CRC serum samples. It was divided into two groups: the training set (130 cases including 31 synchronous liver metastasis cases and 14 heterochronous liver metastasis cases) was used to build up classifiers that were able to distinguish metastatic cases from non-metastatic cases, and the test set (50 heterogeneous liver metastasis cases) for validation. Firstly, we found that serum concentrations of CPA4 in CRC patients were significantly higher than those in healthy controls. Further study demonstrated that serum CPA4 level was higher in the CRC with liver metastasis group than in CRC without liver metastasis group. And the high CPA4 level was correlated with Distant metastasis, Lymph node involvement, Stage and poor survival. By using Logistic regression analysis, we identified that the expression of CPA4 and lymph node involvement were the significant predictors for detecting liver metastasis.

(The odds ratio were 181.44 and 30.677, *p* < 0.05). Leave-one-out method has been employed to validate the efficacy of candidate markers in predicting cancer metastasis [2, 20]. This analysis revealed that CPA4 combining with Lymph node metastasis (N) achieved the most satisfactory sensitivity (95.6%) and specificity (91.8%). The area under the ROCs curve for the detection of CRC liver metastasis by CPA4 and N was 0.855 and 0.815, respectively. When we combined these two markers, ROC was 0.961. Meanwhile, we also got a discriminant equation for the prediction in an independent test set. Moreover, of the 50 heterochronous metastatic cases, this combination could correctly classify 49 cases. Kaplan–Meier survival analysis revealed a correlation between higher CPA4 expression levels and shorter overall survival times. To our knowledge, this is the first study demonstrating that CPA4 expression was closely related to hepatic metastasis and poor prognosis of CRC. We speculated the possible reasons for the contribution of CPA4 to colon cancer liver metastasis. For one thing, CPA4 could degrade extracellular matrix and facilitate cancer cell invasion. For another, CPA4 was highly stained around micro-vessels area, and it is possible to contribute to tumor angiogenesis. Lastly, CPA4 could also be secreted to targeted organs, and induce the pre-metastatic niche formation.

In conclusion, this study firstly shows that CPA4 is highly expressed in human CRC tissues, which is closely related to hepatic metastasis and poor prognosis of CRC. Furthermore, serum CPA4 level combining with Lymph node metastasis may be used as accurate predictors of liver metastasis in colorectal cancer.

## MATERIALS AND METHODS

### Clinical samples and evaluation of immunostaining

Two different commercial tissue arrays were constructed by Shanghai Biochip Co. Ltd. as described previously. One contains 100 cases of colon cancer patients with matched adjacent normal tissues, and another contains 90 cases of rectal cancer patients with matched adjacent normal tissues, respectively. For all the specimens, clinicopathological information (age, gender, pathology, differentiation, TNM stage, and follow-up data) was available. The expression of CPA4 in the tissues was evaluated by immunohistochemical staining with specific antibodies. Standard Avidin-biotin complex peroxidase immunohistochemical staining was performed. Briefly, after deparaffinizationin xylene and graded alcohols, heated antigen retrieval was done in citrate buffer (10 mmol/L pH 6.0) by water-bath kettle heating for 30 min. Endogenous peroxidase was blocked in 0.3% hydrogen peroxide for 10 min. Nonspecific binding was blocked by incubation in 10% normal animal serum for 10min. Sections were incubated at 4°C for 24 h with primary antibodies including polyclonal antibody against anti-CPA4 (Sigma-Aldrich). Immunohistochemistry on the TMAs was graded semiquantitatively considering both staining intensity and percentage of positive tumor cells by two pathologists blinded to the clinicopathologic variables. Protein expression levels were scored by staining intensity and the percentage of immunoreactive cancer cells. The staining intensity was arbitrarily scored on a scale of four grades: 0 (no staining of cancer cells), 1 (moderate or strong staining) and the percentage of positive cells was scored as following: 0 (0%), 1 (1–10%), 2 (10–50%), 3 (> 50%). CPA4 staining positivity was determined using this formula: overall score = positive percentage score × intensity score. Overall score = positive percentage score × intensity score. Next, Raw scores can be converted to standard scores. Colorectal cancer tissues that registered levels 0 and 1 were defined as negative for expression, whereas samples at levels 2 or 3 were defined as positive.

### Measurement of human serum CPA4 level

For ELISA study, preoperative peripheral blood samples were obtained from 180 colorectal cancer patients during 2006–2008 including 31 CRC patients with Synchronous liver metastasis when it had been detected by computed tomography (CT) scan, ultrasonography, or by surgery at the time of initial diagnosis; and 64 CRC ones with heterochronous liver metastasis when liver metastases, confirmed by CT scan or ultrasonography, occurred after resection of the primary tumor. For all the specimens, clinicopathological information (age, gender, differentiation, and TNM stage) was available. At last, 180 specimens were divided into two groups: the training set (130 cases) was used to build up classifiers that were able to distinguish metastatic cases from nonmetastatic cases, and the test set (50 heterogeneous liver metastasis cases) for further validation. 130 specimens of healthy individuals were donated on a voluntary basis. The study was approved by the medical ethics committee of Cancer Hospital, CAMS. CPA4 (SEF317Hu, Cloud-Clone Corp.) concentrations were determined by means of an enzyme linked immunosorbent assay (ELISA) method according to the manufacturer's instructions. The range of the immunoassays for CPA4 was 156 to 10000 pg/mL. Each serum sample was run in duplicate. Briefly, 100 ml of serum (1:5 dilution) were placed into each well of the ELISA plate and incubated for 45 min at 37°C. The plates were washed four times with buffer and incubated with 100 ml of detection antibody at 37°C for 45 min. After four washes, the plates were incubated with substrate solution for 15 min at room temperature, then the reaction was stopped and the plates were read by a spectrophotometer at wavelength 450 nm. A standard curve was generated with the provided standards and used to calculate the quantity of CPA4 in each serum sample.

### Statistical analysis

The SPSS 15 software package (SPSS, Inc., Chicago, IL) was used for statistical analysis. The CPA4 expression was first analyzed as continuous numeric data and the mean staining intensity between the primary tumors and corresponding adjacent normal tissues was compared with a two-sided *t*-test. According to an optimal cutpoint, CPA4 level was analyzed as a dichotomous variable for further evaluation. The association between the markers and clinicopathologic features was analyzed using χ2-test or two-sided *t*-test as appropriate. All possible combinations of these factors were used to build up classifiers that were able to distinguish metastatic cases from non-metastatic cases were examined using Leave-one-out validation model. The discriminant equation and the cut-point of the most satisfactory combination were then used to predict the probability of metastasis in an independent test. The survival rates were assessed by the Kaplan-Meier method and compared by the log-rank test. Statistical significance was set at *P* < 0.05 (two-tailed). Receiver operating characteristics (ROC) curves were generated to compare the predictive sensitivity and specificity, and the area under the curve (AUC). The survival rates were assessed by the Kaplan–Meier method and compared by the log-rank test. Statistical significance was set at *P* < 0.05 (two-tailed).
